# Altered fractional amplitude of low-frequency fluctuations in the superior temporal gyrus: a resting-state fMRI study in anxious depression

**DOI:** 10.1186/s12888-023-05364-w

**Published:** 2023-11-16

**Authors:** Peng Zhao, Xinyi Wang, Qiang Wang, Rui Yan, Mohammad Ridwan Chattun, Zhijian Yao, Qing Lu

**Affiliations:** 1 Department of Medical Psychology, Nanjing Drum Tower Hospital, Affiliated Hospital of Medical School, Nanjing University, Nanjing, China; 2 School of Biological Sciences & Medical Engineering, Southeast University, Nanjing, China; 3 Child Development and Learning Science, Key Laboratory of Ministry of Education, Southeast University, Nanjing, China; 4 Department of Psychiatry, Affiliated Brain Hospital of Nanjing Medical University, Nanjing, China; 5 Nanjing Brain Hospital, Medical School of Nanjing University, Nanjing, China

**Keywords:** Anxious depression, Fractional amplitude of low-frequency fluctuation (fALFF), Resting-state functional magnetic resonance imaging (rs-fMRI), Right superior temporal gyrus (STG)

## Abstract

**Background:**

Anxious depression, which is a common subtype of major depressive disorder, has distinct clinical features from nonanxious depression. However, little is known about the neurobiological characteristics of anxious depression. In this study, we explored resting-state regional brain activity changes between anxious depression and nonanxious depression.

**Method:**

Resting-state functional magnetic resonance (rs-fMRI) imaging data were collected from 60 patients with anxious depression, 38 patients with nonanxious depression, and 60 matched healthy controls (HCs). One-way analysis of variance was performed to compare the whole-brain fractional amplitude of low-frequency fluctuation (fALFF) in the three groups. The correlation between the fALFF values and the clinical measures was examined.

**Results:**

Compared with those of HCs, the fALFF values in the left superior temporal gyrus (STG) in patients with anxious depression were significantly increased, while the fALFF values in the left middle temporal gyrus (MTG), left STG, and right STG in patients with nonanxious depression were significantly increased. Patients with anxious depression showed reduced fALFF values in the right STG compared with patients with nonanxious depression (*p* < 0.001, corrected). Within the anxious depression group, fALFF value in the right STG was positively correlated with the cognitive disturbance score (*r* = 0.36, *p* = 0.005 corrected).

**Conclusion:**

The bilateral STG and left MTG, which are related to the default mode network, appear to be key brain regions in nonanxious depression, while the right STG plays an essential role in the neuropathological mechanism of anxious depression.

## Background

Anxious depression is a clinical subtype of major depressive disorder (MDD) that presents with a high cooccurrence of anxiety-related symptoms [1]. It is estimated that approximately 50 − 80% of MDD patients have a comorbid anxiety disorder or syndromal anxiety symptoms [2–4].

Anxious depression is commonly defined using either syndromal criteria [5] (i.e., cooccurring MDD and at least one anxiety disorder diagnosis) or dimensional criteria [6, 7] (i.e., MDD diagnosis and anxiety/somatization factor score for 17 items on the Hamilton Rating Scale for Depression (HAMD-17) ≥ 7) [8]. In China, since MDD patients have a tendency have depression experiences with significant somatic symptoms [9], the dimensional criteria used to define anxious depression might be more suitable for clinical research in this particular subtype. In comparison with patients with nonanxious depression, patients with anxious depression have more severe somatic symptoms, a lower remission rate, a higher relapse rate or more recurrent episodes, more frequent suicidal ideations, a poorer response to antidepressants, more frequent and intense medication side effects, and a worse prognosis [10]. Although anxious depression is a common illness that leads to serious social and economic burdens [11, 12], there is less information regarding the neurobiological characteristics of anxious depression.

Previous neuroimaging studies have reported both structural and functional alterations in the cortical-limbic circuit, which are involved in emotional and cognitive regulation in patients with anxious depression [13–15]. A study that examined cortical and white matter alterations in MDD patients with comorbid generalized anxiety disorder (GAD) revealed thinning of the right medial orbitofrontal and fusiform gyri, left temporal pole, and lateral occipital cortices [13]. In a task-state functional magnetic resonance imaging (fMRI) study, patients with MDD and anxiety comorbidity who completed the parametric go/no-go (PGNG) test had higher activation in the anterior dorsolateral prefrontal cortex, hippocampus, and caudate within the cognitive control network than controls [14]. In a resting-state fMRI (rs-fMRI) study, Andreescu et al. found stronger functional connectivity (FC) in the posterior regions of the default mode network (DMN) and lower FC in the anterior regions of the DMN in patients with anxious depression than in patients with nonanxious depression [15]. Additionally, patients with anxious depression demonstrated reduced FC between the right centromedial/laterobasal part of the amygdala and right middle frontal gyrus relative to patients with nonanxious depression [16]. Although FC can be used to identify network and regional changes in anxious depression, it can not be used to explain the specific changes and regions that are involved in the primary deficits of the disease. Thus far, to our knowledge, there are only two rs-fMRI studies of brain activity in anxious depression [17, 18]. In the first study, it was observed that, compared to both remitted depression patients and healthy controls (HCs), patients with anxious depression exhibited an increase in the activity of the right dorsal anterior insula, while the activity of the bilateral lingual gyrus decreased [17]. However, the control group comprised remitted depression patients, and therefore, the results could not distinguish between patients with anxious depression and those with nonanxious depression. The second study previously reported that patients with anxious depression exhibited reduced amplitude of low-frequency fluctuation (ALFF) values in the right orbital part of the middle frontal gyrus relative to patients with nonanxious depression [18]. Nevertheless, the ALFF index is sensitive to physiological noise, which might reduce the sensitivity and specificity of spontaneous brain activity.

Fractional ALFF (fALFF) assessment is an advanced technique based on blood oxygen level-dependent (BOLD) fMRI signal intensity of a resting brain. fALFF examinations reduce nonspecific physiological artefacts in rs-fMRI and are thus highly sensitive and specific for detecting spontaneous neuronal activity [19]. In the past, the fALFF index was widely used to detect spontaneous neural activity in patients with depression [20, 21].

The DMN comprises the medial prefrontal cortex, posterior cingulate gyrus, praecuneus, bilateral inferior parietal lobule, middle temporal gyrus (MTG), superior temporal gyrus (STG), inferior temporal gyrus (ITG), and other brain regions [22, 23]. The DMN also participates in various psychological cognitive activities, such as spontaneous thinking, episodic memory, and emotional processing [24, 25]. A meta-analysis of fMRI studies of MDD patients found increased activity in brain regions related to the DMN [26]. However, patients with anxiety disorders exhibit contrasting brain activity patterns within the DMN relative to patients with MDD. For instance, individuals with anxiety disorders might display decreased or underactive functioning of the DMN [22].

Based on the different brain activity characteristics of anxiety disorder and MDD in the DMN, we hypothesized that brain activity would decrease in the regions related to the DMN in patients with anxious depression compared to that in patients with nonanxious depression.

## Methods

### Participants

One hundred and eleven treatment-naive patients with first-episode MDD were enrolled from Nanjing Brain Hospital and Nanjing Drum Tower Hospital. The inclusion criteria for all patients were as follows: (1) a diagnosis of MDD according to the Diagnostic and Statistical Manual of Mental Disorders, 4th edition (DSM-IV) [27]; (2) diagnosis of MDD confirmed by a psychiatrist using the Mini-International Neuropsychiatric Interview(MINI) [28]; (3) right-handed Han Chinese; (4) age range of 18 to 55 years; and (5) a total score greater than 17 on the 17-item Hamilton Depression Rating Scale (HAMD-17).

All patients were grouped into anxious depression and nonanxious depression groups according to the anxiety/somatization subscale items of the HAMD-17. Anxious depression was defined as a score of ≥ 7 on the anxiety/somatization factors and nonanxious depression was classified as a score of < 7 [6–8, 10]. The anxiety/somatization factors included general somatic symptoms, gastrointestinal somatic symptoms, hypochondriasis, insight, psychic anxiety, and somatic anxiety [8, 29]. To obtain more detailed clinical characteristics, all patients were evaluated with the Hamilton Anxiety Rating Scale (HAMA) [30].

Sixty-two HCs, matched for sex, age, and education years, were recruited via advertisements. The MINI was used to assess HCs to confirm that they had no history of mental illnesses. All HCs were right-handed Han Chinese.

The exclusion criteria for all participants were as follows: (1) a family history of mental disorders in first-degree relatives; (2) serious physical illness or organic brain diseases, such as nervous system diseases and traumatic brain injury; (3) a history of alcohol or substance abuse; (4) current pregnancy or breastfeeding; (5) contraindications of MRI; (6) use of antidepressants, psychotherapy, transcranial magnetic stimulation and/or electroconvulsive treatment; and (7) comorbidity with other neurological disorders and mental disorders, such as bipolar disorder, schizophrenia or substance addiction.

Psychological evaluation and magnetic resonance imaging were performed on all patients at Nanjing Brain Hospital. This study was approved by the Research Ethics Review Board of Nanjing Brain Hospital (grant number 2011-KY-027). All participants were informed about the details of the experiment, and informed consent was obtained.

### MRI acquisition and preprocessing

MRI images were collected at the Nanjing Brain Hospital using a 3T Siemens Verio scanner with an eight-channel radio frequency coil. During the MRI scan, all participants were instructed to lie in the supine position and keep their eyes closed, to stay awake and to keep their heads still. Resting-state fMR images were acquired using gradient-recalled echo-planar imaging (GRE-EPI) with the following parameters: repetition time (TR) = 3000 ms, echo time (TE) = 40 ms, matrix = 64 × 64, field of view (FOV) = 240 mm×240 mm, flip angle (FA) = 90°, voxel size = 3.75 × 3.75 × 4 mm^3^, slice = 32, thickness = 4 mm, and volumes = 133. Structural MRI 3D T1-weighted images were acquired using a magnetization-prepared rapid acquisition gradient-echo sequence. The parameters for T1-weighted images were TR = 1900 ms, TE = 2.48 ms, matrix size = 256 × 256, FOV = 250 mm×250 mm^2^, FA = 9°, slice = 176, and thickness = 1 mm.

The processing of fMRI data was performed using MRIcroN (http://www.mricro.com) and the Data Processing Assistant for Resting-State fMRI (DPARSF)(http://www.restfmri.net/forum/DPARSF) toolbox. The raw MRI data were converted from DICOM format into NIFTI format using MRIcroN software. The first six functional volumes were discarded to remove the scanner start-up noise. Then, slice-timing correction and head-motion correction were performed on all the remaining images. Framewise displacement (FD) was calculated according to the work of Power et al. [31]. To minimize the influence of head displacement, ten patients with more than 2 mm movement in any direction and 2° of angular motion during the fMRI scan were excluded from the study. Images were then coregistered to the corresponding high-resolution T1 anatomical images, which were transformed into Montreal Neurological Institute (MNI) space. Afterwards, functional images were resampled to 3 × 3 × 3 mm^3^ voxels and smoothed with a 4-mm full-width, half-maximum Gaussian kernel. Linear detrending and temporal bandpass filtering (0.01–0.08 Hz) were performed. Five participants, including three patients and two HCs, were excluded because of abnormal brain signals.

### fALFF analysis

To investigate regional activity, the grey matter template from the DPARSF toolbox was utilized to compute the whole-brain voxelwise fALFF. The whole brain comprised a total of 271,633 voxels (61 × 73 × 61 voxels). The fALFF maps for each individual were calculated using the DPARSF toolbox. The fALFF for each voxel was defined as the ratio of the power of low-frequency fluctuations (0.01–0.08 Hz) to that of the entire detectable frequency range. fALFF values were considered more sensitive for detecting spontaneous brain activity than ALFF values. The fast Fourier transform was applied to convert the time series into the frequency domain.

### Statistical analysis

We evaluated the demographic characteristics among the three groups and clinical variables between patients with anxious depression and nonanxious depression and the distributions of all demographic and clinical variables were analysed. We used the chi-squared test to compare group differences in sex. For continuous variables, we used a one-way analysis of variance (ANOVA) or two-sample *t* test to compare variables whose variance was homogenous, while a nonparametric test was employed to compare the remaining variables.

ANOVA was used to compare fALFF values among the three groups while controlling for age, sex and education. Additionally, a post hoc *t* test was utilized to compare each pair of groups based on the observed significant clusters among the three groups. The corrected threshold was determined by AlphaSim correction with a threshold of *p* < 0.001. AlphaSim correction was performed using the Resting-State fMRI Data Analysis Toolkit (REST) toolbox. The minimal number of voxels (*k*) in a cluster was 13 for ANOVA, while *k* was 2 for post hoc analysis.

### Relationships with clinical variables

To examine the correlation between brain functional abnormalities and clinical variabilities in the anxious depression group, we performed correlation analyses. The fALFF values involved in correlation analyses were the mean fALFF values within the 4-mm radius sphere centered at the peak of significantly different clusters between anxious depression and nonanxious depression patients. The clinical variables included the total HAMD score, HAMA score, duration of illness, and five-symptom factor scores. The five-symptom factor scores was defined as the total score for the corresponding items in the HAMD, including the anxiety/somatization factor, retardation factor, cognition disturbance factor, hopelessness factor, and sleep disturbance factor. The correlation between significant fALFF values and these clinical variables was examined using *Pearson* correlation analysis. The significance level was set at *p* < 0.0063(Bonferroni multiple comparisons correction *p* < 0.05/8).

## Results

### Demographic and clinical data

Sixty patients with anxious depression, 38 patients with nonanxious depression, and 60 HCs were selected for data analysis. The demographic characteristics and clinical variables of the participants are shown in Table 1. There were no significant differences in sex, age, years of education, or FD among the three groups. The duration of illness between the anxious depression and nonanxious depression groups did not differ significantly. The HAMD-17 score, anxiety/somatization factor score, and HAMA score of the anxious depression group were significantly higher than those of the nonanxious depression group, while the HAMD-17 score minus the anxiety/somatization factor score showed no difference between the two groups.

**Table 1:** Demographic and clinical characteristics of all subjects

Variables(mean ± SD)	AnD	NAnD	HCs	*t*/F	*p* value
	(n = 60)	(n = 38)	(n = 60)		
Sex(male/female)	28/32	19/19	34/26	1.233	0.540^#^
Age(years)	33.53 ± 8.59	31.50 ± 8.97	33.55 ± 9.21	0.757	0.471^*^
Education lever(years)	13.72 ± 2.96	14.13 ± 3.02	14.60 ± 1.72	4.044	0.132^∆^
Duration of illness(month)	7.45 ± 9.50	7.87 ± 9.78		0.044	0.834^**^
HAMD-17	26.35 ± 4.78	20.87 ± 3.23		6.768	0.000^**^
Anxiety/somatization factor	9.25 ± 1.65	5.03 ± 1.05		15.454	0.000^**^
Cognitive disturbance factor	4.77 ± 2.35	4.45 ± 2.09		0.684	0.495^**^
Retardation factor	8.17 ± 1.51	7.53 ± 1.84		1.878	0.063^**^
Weight factor	0.95 ± 0.89	0.76 ± 0.82		1.043	0.300^**^
Diurnal variation factor	0.50 ± 0.72	0.53 ± 0.73		-0.175	0.861^**^
Sleep disturbance factor	3.93 ± 1.76	3.76 ± 2.06		0.436	0.664^**^
Hopelessness factor	5.25 ± 2.03	4.92 ± 2.57		0.668	0.507^**^
HAMD-17-anxiety/somatization Factor	17.10 ± 4.15	15.84 ± 3.16		1.567	0.120^**^
HAMA	25.72 ± 7.29	16.21 ± 3.93		8.360	0.000^**^
FD	0.11 ± 0.06	0.11 ± 0.07	0.11 ± 0.06	0.068	0.93^*^

Abbreviations: AnD, anxious depression; NAnD, nonanxious depression; HCs, healthy controls; SD, standard deviation; HAMD-17, 17-item Hamilton Depression Rating Scale; HAMA, Hamilton Anxiety Scale; FD, frame-wise displacement

# *p* values for chi-square test

* *p* values for one-way ANOVA.

^∆^ *p* values for nonparametric test

** *p* values for two-sample *t*-tests

#### fALFF

**Fig. 1:**
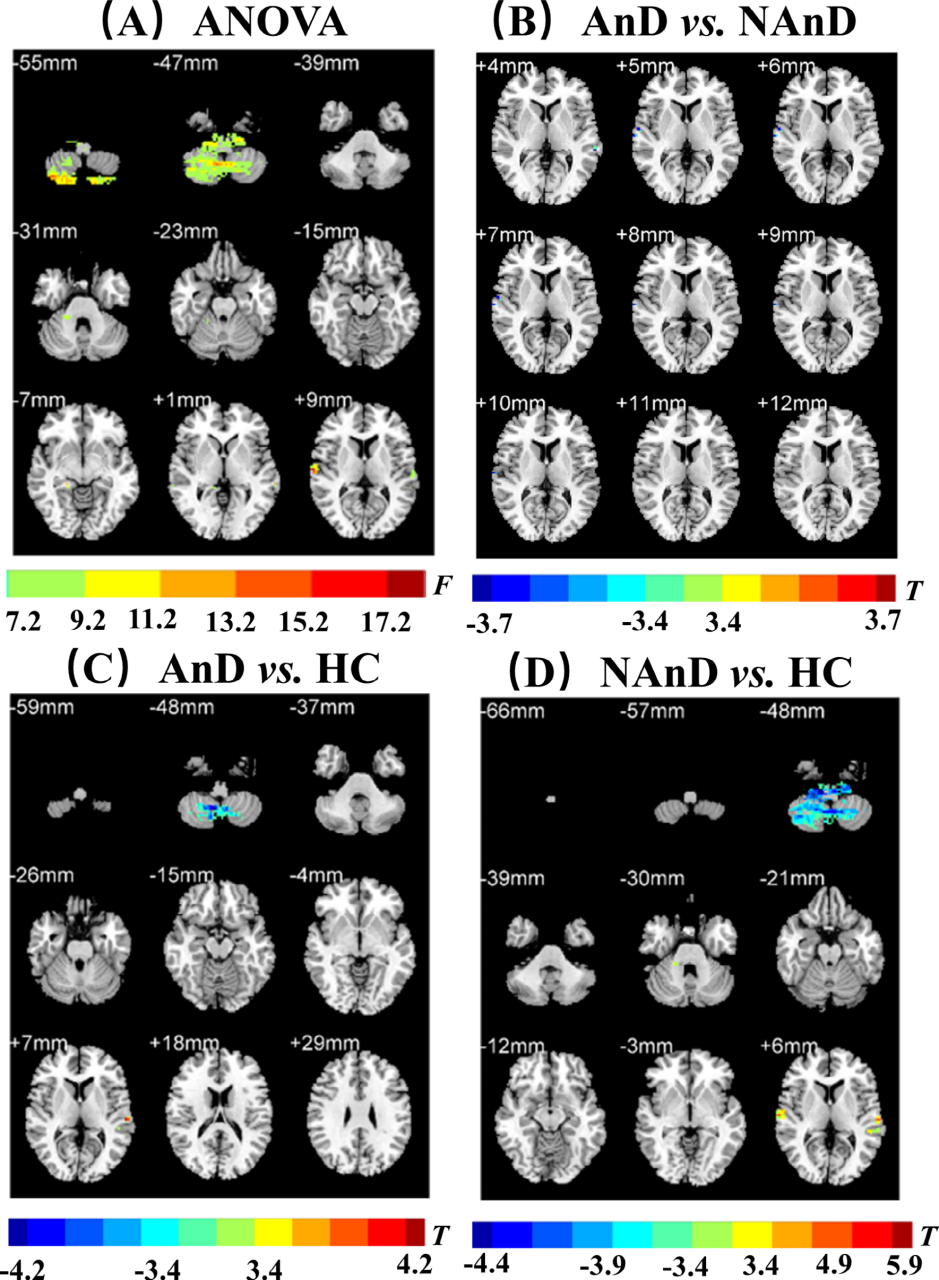
Clusters showing significantly differences. **A**: Clusters showing the significantly differences in the fALFF among the three groups. **B**: Clusters showing the significantly differences in the fALFF between AnD group and NAnD group. **C**: Clusters showing the differences in the fALFF between AnD group and HCs. **D**: Clusters showing the differences in the fALFF between NAnD and HCs. The color bar signifies the F or *t* value. Abbreviations: AnD, anxious depression; NAnD, nonanxious depression; HCs, healthy controls; ANOVA, analysis of variance; fALFF, fractional amplitude of low-frequency fluctuation

**Group differences**. There were five clusters with significant differences among the three groups. The observed clusters were named according to their peak coordinate locations: the right STG, the left STG, the left MTG, right cerebellar lobule 4 and 5, and right cerebellar lobule 7b (*p* < 0.001, *k* > 13, *p* < 0.05 corrected for multiple comparisons with AlphaSim)(see Table 2; Fig. 1A).

**Table 2:** Brain areas with fALFF difference among all groups

Cluster names	Peak MNI coordinates			Voxels^*^	F/*t* value
	x	y	z		
**Three groups comparison**					
R STG	69	-15	9	36	17.848^a^
L STG	-63	-21	6	18	16.015^a^
L MTG	-66	-33	0	18	11.486^a^
R cerebelar lobule 4 and 5	21	-39	-27	16	13.790^a^
R cerebelar lobule 7b	42	-72	-54	872	13.051^a^
**Two-group pairs comparison**					
AnD < NAnD					
R STG	60	-15	9	4	-3.700^b^
AnD <HCs					
R cerebelar lobule 7b	42	-72	-54	20	-3.680^b^
R cerebelar lobule 8	33	-54	-57	4	-3.493^b^
R cerebelar lobule 9	9	-57	-48	87	-4.188^b^
L cerebelar lobule 9	-18	-51	-51	4	-3.727^b^
AnD >HCs					
L STG	-60	-24	6	6	4.292^b^
NAnD >HCs					
R cerebelar lobule 4 and 5	21	-39	-27	12	4.927^b^
L MTG	-66	-33	0	18	4.874^b^
L STG	-63	-21	6	16	5.443^b^
R STG	69	-15	9	34	6.103^b^

Abbreviations: Anatomical Automatic Labeling; MNI, Montreal Neurologic Institute; AnD, anxious depression; NAnD, nonanxious depression; HCs, healthy controls; fALFF, fractional amplitude of low-frequency fluctuation; STG, Superior Temporal Gyrus; MTG, Middle Temporal Gyrus; L = left; R = right

^a^The F statistical value

^b^The *t* statistical value

* The minimal number of voxels (*k*) in a cluster was 13 for ANOVA, while *k* was 2 for post hoc analysis

#### fALFF

Anxious depression patients vs. nonanxious depression patients. Relative to that of nonanxious depression patients, the fALFF value of the right STG was significantly lower in anxious depression patients (*p* < 0.001, *k* > 2, *p* < 0.05 corrected for multiple comparisons with AlphaSim)(see Table 2; Fig. 1B).

#### fALFF

Anxious depression patients vs. **HCs**. In contrast to those of HCs, the fALFF values of right cerebellar lobule 7b, right cerebellar lobule 8, right cerebellar lobule 9, and left cerebellar lobule 9 were significantly lower in patients with anxious depression, while the fALFF value of the left STG was significantly higher (*p* < 0.001, *k* > 2, *p* < 0.05 corrected for multiple comparisons with AlphaSim)(see Table 2; Fig. 1C).

#### fALFF

Nonanxious depression patients vs. **HCs**. Compared with HCs, the patients with nonanxious depression had higher fALFF values in right cerebellar lobule 4 and 5, the left MTG, the left STG, and the right STG(*p* < 0.001, *k* > 2, *p* < 0.05 corrected for multiple comparisons with AlphaSim)(see Table 2; Fig. 1D).

### Correlational analysis between fALFF values and clinical data

**Fig. 2:**
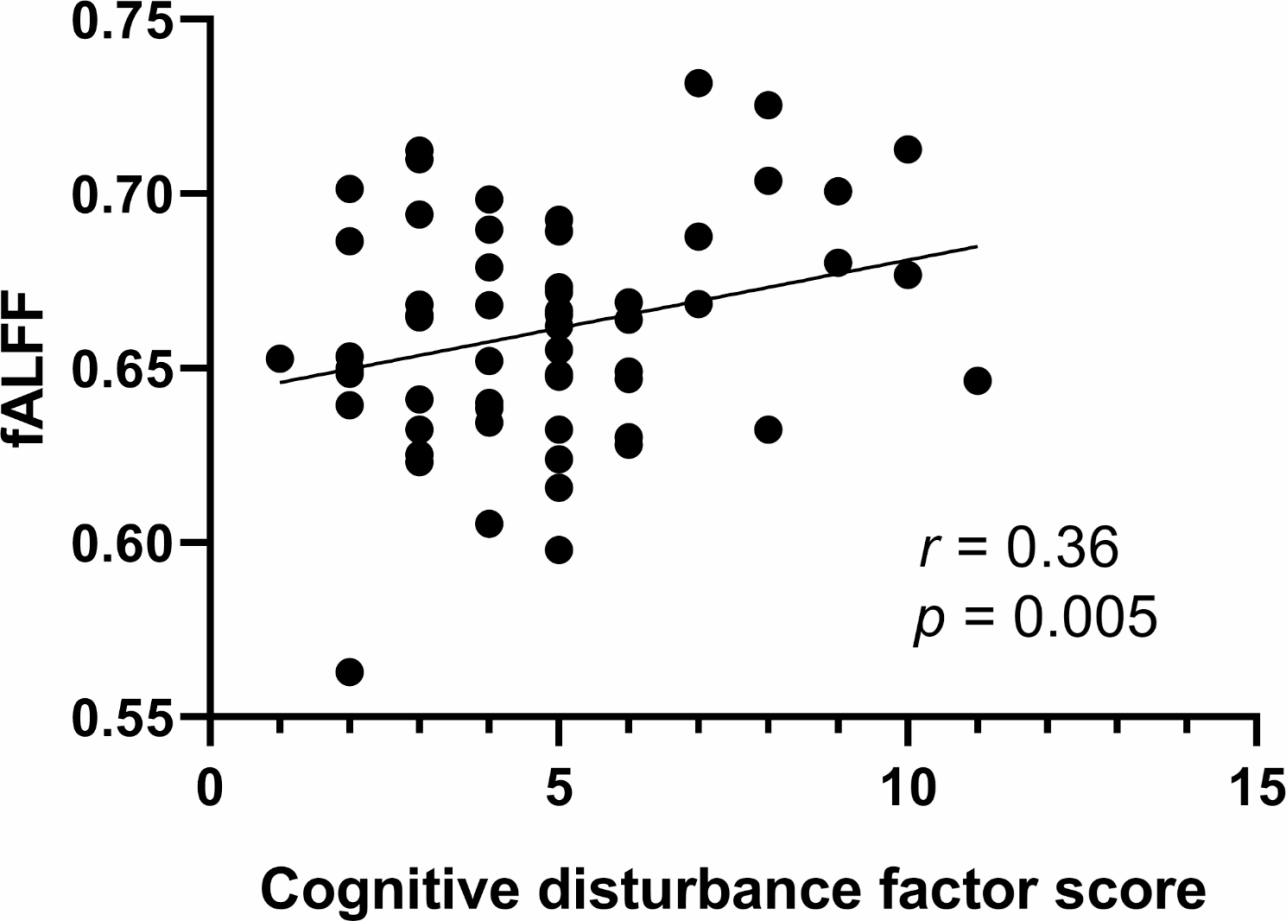
Positive correlation between the fALFF values of STG in AnD group with cognitive disturbance factor scores. Abbreviations: fALFF, fractional amplitude of low-frequency fluctuation; AnD, anxious depression; STG, Superior Temporal Gyrus

There was a significantly positive correlation between the fALFF value of the right STG and the cognitive disturbance factor score in the anxious depression group (*r* = 0.36, *p* = 0.005 corrected for multiple comparisons with Bonferroni correction)(see Fig. 2). All the corrected and uncorrected results are shown in Table 3.

**Table 3:** Correlation between the fALFF value of right STG in AnD group and the total score of HAMD-17, factor score, total score of HAMA, and duration of illness

	Correlation coefficient	*p* value
HAMD-17	0.2831	0.0284
Anxiety/somatization factor	0.2493	0.0547
Retardation factor	0.2654	0.0404
Cognitive disturbance factor	0.3553	0.0053*
Hopelessness factor	-0.2980	0.0208
Sleep disturbance factor	-0.0164	0.9011
HAMA	0.2471	0.0569
Duration of illness	0.2493	0.0547

Abbreviations: AnD, anxious depression; HAMD-17, 17-item Hamilton Depression Rating Scale; HAMA, Hamilton Anxiety Scale; STG, Superior Temporal Gyrus

* Correlation remains significant after Bonferroni multiple comparisons correction (*p* < 0.05/8 = 0.0063)

## Discussion

In the present study, patients with anxious depression exhibited significantly lower fALFF values in the right STG in contrast to patients with nonanxious depression and significantly higher fALFF values in the left STG compared to HCs. In addition, the fALFF values of the left MTG, left STG, and right STG were significantly higher in patients with nonanxious depression than in HCs. Furthermore, the fALFF values of the right STG positively correlated with the cognitive disturbance factor score in patients with anxious depression.

Our findings are in line with previous structural magnetic resonance studies that reported decreased grey matter volume in the STG and MTG in individuals with depression [32, 33]. Moreover, fMRI studies have identified brain activity alterations in the MTG and STG in patients with depression [34, 35]. Collectively, these results indicate that the MTG and STG play a role in the neuropathogenesis of depression. Both the MTG and STG are integral components of the DMN, which is particularly active during resting states [24] and is closely related to self-reference, rumination, spontaneous thinking, episodic memory, cognitive control, and emotional processing [36, 37]. Notably, the patients with depression exhibited more psychological activity of self-reference [38]. A meta-analysis of fMRI studies demonstrated increased activity in brain regions linked to the DMN in MDD patients [26]. Moreover, subtype analysis of depression further supported these findings, showing that the fALFF value in the STG of patients with anxious depression was significantly higher than that of HCs [39], which was consistent with our results.

The right STG participates in emotional and high-level cognitive functions such as emotional attention [40], social cognition [41], spatial perception [42], and exploration [43]. Previous structural MRI studies have confirmed the role of the right STG in the pathology of depression [44–46]. fMRI studies also revealed an association between the STG and depression [46–48]. Moreover, both current and remitted MDD patients exhibited morphologic changes in subregions of the STG [33]. Li et al. found that the regional homogeneity value of the right STG was significantly higher in patients with depression than in HCs [49]. In addition, the right STG is closely related to the generation of anxiety. Patients with social anxiety disorder and GAD had lower FC in the right and bilateral STG, respectively [50, 51]. Patients with severe anxiety disorder showed significantly reduced FC in regions of the DMN, including the right STG [23]. From these studies, it can be inferred that the function of the right STG increases in depression and decreases in anxiety. Anxious depression could be considered MDD with the presence of anxiety, which might explain why the fALFF value of the right STG was significantly lower in patients with anxious depression than in patients with nonanxious depression.

In our results, the fALFF value of the right STG had a positive correlation with the cognitive disturbances score in patients with anxious depression. Notably, the DMN is the most active network at rest in MDD patients and is related to negative rumination [52]. Furthermore, excessive self-referential processing, which plays a critical role in cognition, was activated in DMN structures, including the STG [53]. Therefore, higher a fALFF value in the right STG of patients with anxious depression might lead to increased self-reference and rumination activities, ultimately contributing to cognitive impairment.

In our study, the fALFF values of right cerebellar lobule 7b, right cerebellar lobule 8, and bilateral cerebellar lobule 9 decreased significantly in patients with anxious depression compared to those of HCs. Cerebellar lobules 7b, 8, and 9 are part of the visual network and are involved in advanced cognitive and emotional activities [54, 55]. In addition, cerebellar lobule 7b is part of the auditory network and salience network [55]. Cerebellar lobule 8 is a component of the sensorimotor network and is activated during pain-related processes, while cerebellar lobule 9 is related to the DMN [55]. Furthermore, there was strong rs-FC among cerebellar lobule 8, cerebellar lobule 9 and the amygdala [55]. Since the cerebellar lobules 7b, 8, and 9 are associated with the salience network, amygdala, and DMN, there is a possibility that reduced activity in these lobules might impair the ability to regulate anxiety.

There were several limitations in the study. First, the sample size was relatively small. Second, this study was cross-sectional study and did not consider the dynamic changes in brain activity during treatment. Future research should include a larger sample size and investigate the dynamic changes in brain activity before and after treatment. Finally, while we identified clusters in the cerebellum, it is essential to highlight the need for a closer examination of the pathology of MDD. Given the cerebellum’s unique physiological characteristics and anatomical location, the signals observed in this region are susceptible to potential confounding factors. Therefore, it is imperative to interpret the cerebellar results with caution, recognizing the complexities of this brain region in the context of MDD. Further investigations into the underlying nature of these signals are warranted in future research to provide a more comprehensive understanding of their significance and potential relevance to the disorder.

## Conclusion

In summary, we used the fALFF index to explore the characteristics of resting-state brain activity in unmedicated patients with anxious depression. Our preliminary findings suggested that the bilateral STG and left MTG might be key brain regions in nonanxious depression. In addition, the right STG appears to play an essential role in the neural basis of anxious depression. The neural activity alterations within the STG might contribute to the emotional and cognitive aspects of anxious depression. Further research is needed to validate and corroborate the roles of these brain regions in nonanxious depression and anxious depression.

## Data Availability

The datasets used and/or analysed during the current study available from the corresponding author on reasonable request.
